# Response to: Commentary: What Is Art Good For? The Socio-Epistemic Value of Art

**DOI:** 10.3389/fnhum.2018.00237

**Published:** 2018-06-07

**Authors:** Aleksandra Sherman, Clair Morrissey

**Affiliations:** ^1^Department of Cognitive Science, Occidental College, Los Angeles, CA, United States; ^2^Department of Philosophy, Occidental College, Los Angeles, CA, United States

**Keywords:** neuroaesthetics, empirical aesthetics, art, cognitive neuroscience, social neuroscience

Skov and Nadal's (2017) commentary on our paper “What is Art Good For? The Socio-Epistemic Value of Art” (Sherman and Morrissey, 2017) provides us with a valuable opportunity to clarify our argument, with special attention to the need for greater and continued collaboration between neuroaesthetics and other disciplines that study the arts.

We are heartened that the commenters concur with our central claim: neuroaesthetics can profitably do, and has not yet done, significant work on the social dimensions of art appreciation and creation. Although a number of studies have taken first steps toward this important work—especially with respect to music, literature, and film—we detail in our paper the many ways in which neuroaesthetics has, indeed, somewhat narrowly focused on understanding beauty and preferences. For example, even work on individual differences between experts and novices, a potentially rich arena for understanding the social development of cognitive capacities relevant to art appreciation, is often done in service of measuring preference or beauty judgments rather than in service of understanding socially-relevant outcomes.

We also agree that the tools of experimental research have mostly been ill-suited to the new questions we pose. Not only, as Skov and Nadal suggest, because some of the relevant technology cannot yet readily go into social settings, but primarily because many experimental designs within neuroaesthetics have not been aimed at capturing the long-term development of skills, capacities, and interactions. Although technological developments—including mobile EEG systems or hyperscanning—may increase accessibility for measuring psychological processes outside the lab, technology *per se* does not solve the problem. Technology is a tool; the issues we identify concern the questions asked, not merely the means used to answer them. Furthermore, contra Skov and Nadal's characterization of our argument, research programs that attempt to capture the experience of art appreciation in its “social context” or that translate current experimental paradigms to a “social setting” are not enough. A fuller exploration of the arts would require neuroaesthetics to understand art as something we *do*. We expressed this using the notion of “social practices,” understood as forms of human cooperative activity that exist within social groups and persist through time.

We demonstrated that thinking of art as a social practice invites a host of questions about how the arts function as cooperative activities, both in particular moments and across time and generations, about what it means to learn to create and appreciate art over a lifetime, and about the cognitive, social, or developmental implications of such practices. Far from discounting the importance of prior work in neuroaesthetics, we suggested that the substantial body of literature on art perception, representation, and valuation be understood as contributing in a relevant, albeit limited, way to our understanding of this more robust conception of the arts. Ours is a call to ask new questions and to locate and frame what we already know in a new way. For example, one may ask how behavioral and physiological outcomes of art appreciation (e.g., being moved, tears, chills, thrills, arousal) indicate self-referential processing and self-understanding; whether and how individuals with more art expertise possess stronger self-reflective skills and/or stronger empathetic tendencies; and how the processes relevant to other-understanding (e.g., perspective-taking, imitation, emotional resonance) may be recruited during art appreciation (for a more extensive list of sample open questions, see Table 2 in Sherman and Morrissey, 2017).

Moreover, we believe Skov and Nadal mischaracterize the role of philosophy in the argumentative structure and strategy of our paper. Our use of philosophical conceptions of the value of art is not a bald assertion that these claims are true, is not based solely on our intuitions, and is not an appeal to authority. Rather, these philosophical conceptions allow us to draw new questions and directions for empirical investigation from a different disciplinary way of thinking. Philosophers' claims and insights often contain or imply testable empirical conjectures. We highlighted contemporary philosophical arguments which suggest that arts appreciation might contribute to the cultivation of self and other-understanding, and demonstrated how empirical evidence may be brought to bear on those theories by reviewing the related neuroaesthetics literature. Hence, we stand by our “peculiar” decision to focus our discussion on these particular skills, as they have demonstrated resonance with philosophical thought *and* with the psychological and neuroscientific literature. We also emphasize that self and other-understanding are *examples*, and do not exhaust the possible social and epistemic values associated with the art practices.

Finally, we strongly resist Skov and Nadal's suspicion of theory's usefulness for neuroaesthetics. Of course there is deep disagreement, philosophical and otherwise, about the nature of art. However, the fact that theorists disagree is not evidence that there is no correct answer, or, more importantly, that theory has no productive role to play with respect to empirical work. We understand the relationship between philosophy and neuroaesthetics as complementary; empirical evidence helps us shape theories, and theory helps inform new and revelatory questions, and helps us interpret and give meaning to our data. It is unclear how any empirical research program could make progress without a theoretical notion of what one is studying and why it matters. For these reasons, we also stand firmly behind our paper as a demonstration of how disciplines may productively collaborate toward a shared understanding of what art is doing in our lives and communities.

We agree that neuroaesthetics can and should do better. Indeed, we hope that by conceptualizing a richer theoretical understanding of art, we will encourage neuroaestheticians to more deeply consider new experimental approaches that highlight the importance of social outcomes.

## Author contributions

All authors listed have made a substantial, direct and intellectual contribution to the work, and approved it for publication.

### Conflict of interest statement

The authors declare that the research was conducted in the absence of any commercial or financial relationships that could be construed as a potential conflict of interest.
